# Species Identification and Antimicrobial Resistance of *Streptococcus* spp. Isolated from Nasal Swabs of Wild Boars in Avellino Province, Southern Italy

**DOI:** 10.3390/ani16111619

**Published:** 2026-05-26

**Authors:** Francesca Paola Nocera, Consiglia Longobardi, Annunziata Romano, Rossana Schena, Nadia Piscopo, Carlo Romei, Valeria Iervolino, Luisa De Martino, Sara Damiano, Roberto Ciarcia

**Affiliations:** Department of Veterinary Medicine and Animal Production, University of Naples Federico II, Via F. Delpino, n. 1, 80137 Naples, Italy; francescapaola.nocera@unina.it (F.P.N.); consiglia.longobardi@unina.it (C.L.); annunziata.romano@unina.it (A.R.); rossana.schena@unina.it (R.S.); nadia.piscopo@unina.it (N.P.); ca.romei@studenti.unina.it (C.R.); valeria.iervolino@unina.it (V.I.); roberto.ciarcia@unina.it (R.C.)

**Keywords:** *Streptococcus* spp., wild boar, nasal microbiota, antimicrobial resistance

## Abstract

From an ecological epidemiology perspective, wildlife nasal cavities may serve as critical ecological niches for the maintenance and spread of bacterial pathogens relevant to both animal and human health. This study investigated *Streptococcus* spp. as the most prevalent genus in the nasal cavities of 82 wild boars (*Sus scrofa*), sampled across six hunting districts in Avellino Province (Southern Italy), assessing the diversity of the colonizing streptococcal populations and their antimicrobial resistance profiles. The sampled population consisted of 43 males (52%) and 39 females (48%). A total of 173 Gram-positive isolates were recovered, with 74 identified as *Streptococcus* spp. by MALDI-TOF MS. These isolates originated from 59 positive swabs (72%; 59/82). *Streptococcus gallolyticus* was the most prevalent species (41%; 30/74), followed by *Streptococcus porcinus* (27%; 20/74) and *Streptococcus suis* (19%; 14/74). No statistically significant geographic association was found across sampling districts (*p* = 0.491), whereas a significant association was found between bacterial species and antimicrobial resistance profiles (*χ*^2^ = 16.70, *p* < 0.001). Specifically, all *Streptococcus porcinus* isolates (100%; 20/20) exhibited resistance to sulfamethoxazole–trimethoprim and tetracycline, while high tetracycline resistance was also noted in *Streptococcus suis* (93%; 13/14) and *Streptococcus gallolyticus* (83%; 25/30). Overall, 69% (51/74) of isolates were classified as MDR and 5% (4/74) as XDR. These findings suggest the potential role of wild boars as reservoirs for antimicrobial-resistant streptococci, emphasizing the need for continuous surveillance under a One Health framework.

## Simple Summary

Wild boars may serve as reservoirs for various bacteria that can affect both livestock and humans. This study focused on the bacterial flora of the nasal cavities of wild boars, especially *Streptococcus* species, which were found to be the most prevalent, to understand their diversity and levels of antimicrobial resistance. Our findings revealed the presence of several important streptococcal species, many of which showed high resistance to commonly used antibiotics like tetracycline. These results highlight the potential role of wild boars in the environmental circulation of resistant bacteria, emphasizing the need for continued monitoring to protect animal and public health.

## 1. Introduction

While the role of livestock in the dissemination of antimicrobial resistance is well-documented [1,2], the epidemiological contribution of wildlife, particularly wild boars (*Sus scrofa*), remains insufficiently characterized and represents a critical knowledge gap [3,4]. Wild boars act as mobile sentinels and potential reservoirs for antimicrobial resistance genes in the environment [3]. Within these hosts, the nasal cavity represents a distinct clinical sampling site, mediating the balance between commensalism and infection. It serves as a primary niche for the colonization and persistence of both commensal and pathogenic species, influencing local immune responses and host susceptibility to respiratory infections [5]. Furthermore, the nasal cavity hosts a complex microbiota, typically dominated by major, stable genera such as *Staphylococcus*, that is essential for defending against infections [5]. However, the nasal microbial composition may exhibit significant diversity; indeed, the genus *Streptococcus* also represents a dominant and highly diverse taxon within this niche [6].

The genus *Streptococcus* currently includes over 100 species, ranging from essential commensals to highly virulent pathogens responsible for significant public health burdens worldwide [7,8,9]. In veterinary medicine, several *Streptococcus* species are of major clinical importance, including *Streptococcus canis* in dogs and cats, *Streptococcus equi* subsp. *equi*, the causative agent of strangles in horses, and *Streptococcus agalactiae*, a major cause of bovine mastitis. In addition, *Streptococcus suis* is a major zoonotic swine pathogen associated with severe systemic conditions such as meningitis, septicemia, arthritis, and endocarditis [10]. Other streptococcal species have also been identified in a wide range of hosts, including birds, aquatic animals, and livestock [11].

Research on *Streptococcus* species in wild boars remains limited compared with the extensive literature available for domestic pigs [12,13]. Nevertheless, several species, including *Streptococcus porcorum* and *Streptococcus suis*, have been isolated from wild boar populations, often in the absence of evident clinical disease. This supports the hypothesis that wild boars may act as asymptomatic reservoirs of potentially zoonotic strains [14]. Molecular surveys detecting bacterial DNA in clinically healthy animals further suggest that streptococcal colonization may occur without associated lesions, contributing to environmental persistence and interspecies transmission [12,15].

Prevalence studies have reported substantial carriage rates in some regions. In northwestern Europe, *Streptococcus suis* was isolated from up to 92% of tonsil samples collected from wild boars, with a proportion of isolates carrying virulence-associated genes [16]. These findings highlight the potential zoonotic relevance of wild boar populations, particularly for individuals with occupational or recreational exposure, such as butchers and hunters. Although wild boars are often asymptomatic carriers, clinical disease has occasionally been reported, including fatal cases of septicemia and meningitis [17]. Population-level investigations have also revealed considerable genetic diversity among wild boar isolates, which frequently differ from the dominant clonal complexes identified in domestic pigs and humans [18]. Consequently, wild boars may represent a localized interface at the wildlife–livestock–human boundary, although the actual spillover risk likely depends on the extent of overlap with agricultural environments [19].

Beyond *Streptococcus suis*, other *Streptococcus* species have occasionally been reported in wild boars, suggesting that this host harbors a broader streptococcal community than currently recognized [18,20]. However, the occurrence of specific species such as *Streptococcus gallolyticus* remains sporadically documented, and their ecological and epidemiological significance at the wildlife–livestock–human interface is still poorly understood [21].

Therefore, this study aimed to characterize the species distribution and antimicrobial resistance profiles of the *Streptococcus* species most frequently isolated from the nasal cavities of healthy wild boars hunted in the Avellino Province (Southern Italy), to expand the currently limited epidemiological data on nasal microbiota and antimicrobial resistance in wildlife. 

## 2. Materials and Methods

### 2.1. Ethics Statement

Ethical approval was not required for this study because the animals were not killed specifically for research purposes. Wild boars were legally harvested in their natural environment by licensed hunters in accordance with the 2025–2026 annual hunting plan authorized by the Province of Avellino (Campania Region, Italy). Samples were collected post-mortem during the official hunting season in compliance with national wildlife management regulations. Informed consent to participate in this research study was obtained by licensed and specialized hunters, who provided wild boar nasal swabs.

### 2.2. Study Area and Sample Collection

#### 2.2.1. Study Area

**Figure 1:**
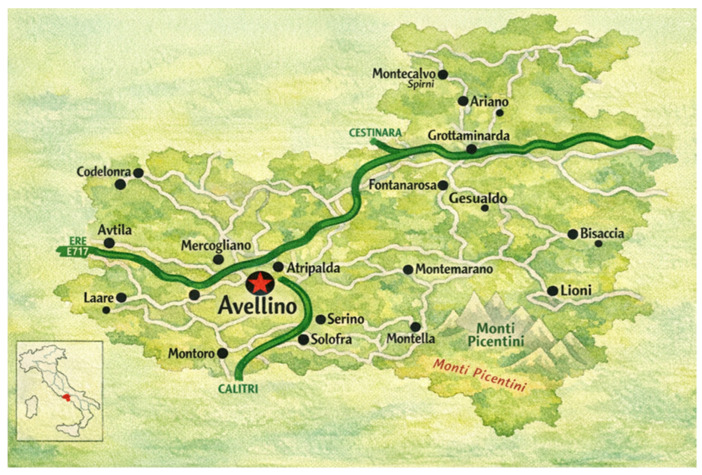
Geographic location of the study area: detailed map of the Avellino Province (Irpinia) located in Campania Region, Southern Italy.

The study was conducted in the Province of Avellino, located in the inland sector of the Campania region, Southern Italy (Figure 1). The Campania region occupies the south-western portion of the Italian Peninsula, covering an area of 13,590 km^2^ and extending along approximately 350 km of coastline on the Tyrrhenian Sea. The Province of Avellino is centered approximately at 40°55′00″ N and 14°47′00″ E and is characterized by predominantly mountainous and hilly terrain, with elevations ranging from about 200 to over 1800 m above sea level, including part of the Apennine chain. The area exhibits high environmental heterogeneity, with extensive forested habitats interspersed with agricultural land, creating a complex mosaic of wildlife habitats. Climatic conditions are typical of inland Mediterranean environments, with significant seasonal thermal variation. This heterogeneous topography, together with the strong connectivity between forested and semi-natural landscapes, provides favorable conditions for the expansion and high population density of wild boar (*Sus scrofa*), promoting frequent interactions with agricultural environments and human settlements.

**Figure 2:**
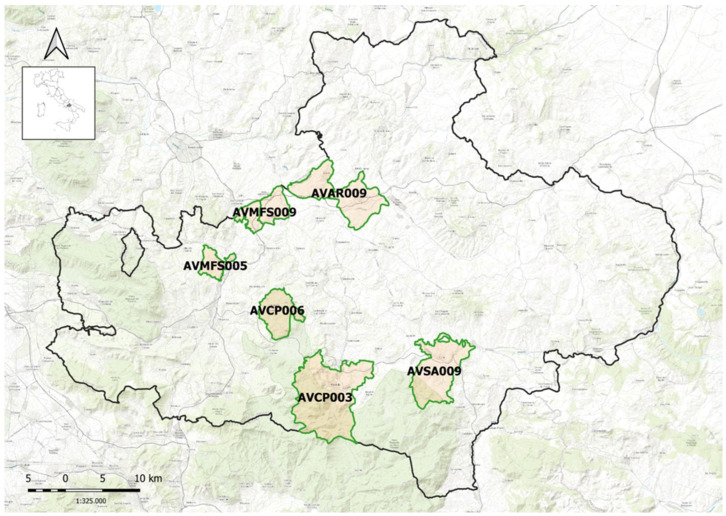
Spatial distribution of the sampling districts within the Avellino Province. Highlighted areas represent the specific hunting districts where wild boar specimens were collected: AVMFS009 Medio Fiume Sabato District (Venticano/Pietradefusi); AVAR009 Arianese District (Bonito-Grotta-minarda); AVCP006 Picentini District (Chiusano); AVMFS005 Medio Fiume Sabato District (Prata di Principato Ultra); AVCP003 Picentini District (Montella); AVSA009 Sant’Angelo District (Lioni).

Specifically, the study focused on wild boar hunting activities across six different hunting districts within the Avellino Province as reported in Figure 2.

#### 2.2.2. Study Design and Sample Collection

This cross-sectional study employed convenience sampling of wild boars harvested by authorized hunting teams during the 2025–2026 hunting season (from 1 October 2025 to 1 January 2026). After hunting, the animals were transported to a central processing site for carcass dressing and sample collection. Nasal swabs were collected from clinically healthy male and female wild boars, weighing between 15 and 160 kg, showing no gross pathological lesions at post-mortem inspection. The age of the hunted animals was estimated based on tooth eruption patterns, and individuals were classified into three age groups: juveniles (0–12 months), subadults (13–36 months), and adults (>36 months). The sampling was performed by inserting and rotating a single sterile swab in both nostrils of each animal. Each swab was placed in Stuart W/O CH transport medium (Aptaca Spa, Asti, Italy) to maintain bacterial viability and prevent desiccation. The specimens were then labeled and transported within 48 h to the laboratory in an insulated icebox containing refrigerant packs, ensuring that the temperature did not exceed 10 °C. Bacteriological analyses were conducted at the Bacteriology Diagnostic Laboratory of the Department of Veterinary Medicine and Animal Production, University of Naples Federico II.

### 2.3. Species Identification and Antimicrobial Susceptibility Testing

Nasal swabs were cultured in parallel on different solid agar media for the isolation of both Gram-positive bacteria (Columbia CNA blood agar and Mannitol Salt Agar) and Gram-negative bacteria (Mac Conkey agar), as well as in Brain Heart Infusion (BHI) broth for enrichment, and incubated aerobically at 37 °C for 24 h. The following day, turbid BHI cultures were subcultured onto the same agar media. All media were purchased from Liofilchem S.r.l. (Teramo, Italy).

Specifically, the isolation of *Streptococcus* spp. was performed using Columbia CNA agar (CNA) supplemented with 5% sheep blood (Liofilchem S.r.l., Teramo, Italy), a selective medium for Gram-positive bacterial isolation. Once bacterial growth was observed on CNA plates, colonies suspected to belong to *Streptococcus* spp. (small, translucent colonies surrounded by alpha- or beta-hemolysis) were preliminarily screened using standard rapid identification techniques, including colony morphology assessment, Gram staining, and catalase testing. Subsequently, isolates were identified to the species level using Matrix-Assisted Laser Desorption/Ionization–Time of Flight Mass Spectrometry (MALDI–TOF MS) using the MALDI Biotyper Sirius System (Bruker Daltonics Inc., Bremen, Germany). For each isolate, analyses were performed in triplicate to ensure reproducibility of the spectral profiles. Spectra were acquired in linear positive ion mode using FlexControl 3.4 software (Bruker Daltonics, Bremen, Germany). Identification was achieved by comparing the acquired spectra with the Bruker MSP database using the Bruker MBT Compass HT software (MBT Compass HT RUO version 5.1.400.9024) with default parameters. Identification accuracy was determined in accordance with the manufacturer’s scoring thresholds. Specifically, scores ≥ 2.00 were considered indicative of high-confidence species identification, while values ranging between 1.70 and 1.99 were used to establish a confident identification at the genus level. Any scores falling below 1.70 were treated as providing no reliable identification. To ensure analytical precision and maintain quality control throughout the process, a bacterial test standard (BTS) (Bruker Daltonics, Bremen, Germany) was employed as the system calibrator. *Streptococcus equi* subsp *zooepidemicus* ATCC^®^ 53698^TM^ and *Staphylococcus aureus* ATCC^®^ 33591^TM^ were included as quality control strains.

Susceptibility testing was performed by agar disk diffusion method on Mueller–Hinton agar plates (Liofilchem S.r.l., Teramo, Italy). The collected isolates were evaluated for their susceptibility to eleven antimicrobial agents belonging to eight different antimicrobial classes. These agents, along with their respective classes, are listed in Table 1. All tested antimicrobial disks were purchased from Liofilchem S.r.l. (Teramo, Italy).

Specifically, isolates were classified as susceptible, intermediate, or resistant to amoxicillin/clavulanic acid (AMC), ampicillin (AMP), penicillin (P), cefalexin (CL), enrofloxacin (ENR), gentamicin (CN), and erythromycin (E) according to the Clinical and Laboratory Standards Institute (CLSI) guidelines [22]. Susceptibility to imipenem (IMI), meropenem (MRP), tetracycline (TE), and sulfamethoxazole–trimethoprim (SXT) was interpreted according to the European Committee on Antimicrobial Susceptibility Testing (EUCAST) guidelines [23]. In addition, the recovered *Streptococcus* species were categorized as multidrug-resistant (MDR), extensively drug-resistant (XDR) and pandrug-resistant (PDR) following the criteria proposed by Magiorakos et al. [24]. For classification purposes, isolates displaying resistance to at least one antimicrobial agent per category were considered. MDR isolates were defined as those resistant to at least one agent in three or more antimicrobial categories, XDR isolates as those resistant to at least one agent in most antimicrobial categories, and PDR isolates as those resistant to all antimicrobial categories tested.

*Streptococcus* spp. isolates were then preserved in 16% *v*/*v* glycerol broth and in Microbank tubes (Pro-Lab Diagnostics, Round Rock, TX, USA) at −80 °C for further investigation.

### 2.4. Data Handling and Descriptive Statistical Analysis

All microbiological and antimicrobial susceptibility results generated by the Microbiological Diagnostic Laboratory were recorded in a Microsoft Excel™ spreadsheet (Microsoft 365, Microsoft Corp., Redmond, WA, USA). For each isolate, the following variables were recorded: sample identification code, sampling district, host sex and body weight, *Streptococcus* species, and antimicrobial resistance profile for each tested antimicrobial agent. Descriptive statistical analyses were performed by calculating absolute frequencies, percentages, and 95% confidence intervals (95% CI) to determine the occurrence of the different *Streptococcus* species and the distribution of antimicrobial resistance phenotypes among recovered isolates. Graphical representations were generated using Microsoft Excel™. The association between categorical variables was assessed using Pearson’s Chi-square test (R × C contingency tables), specifically for the distribution of *Streptococcus* species across sampling districts, the distribution of multidrug resistance phenotypes (MDR/XDR) among bacterial species, and the influence of host age categories (0–12, 13–36, >36 months) on MDR/XDR phenotypes. Cramer’s V coefficient was calculated to estimate the strength of the association where applicable. To analyze the association between *Streptococcus* species and antimicrobial phenotypes, resistance profiles were dichotomized into an ‘MDR/XDR’ group versus a ‘non-MDR’ group to ensure statistical robustness. Standardized residuals (*SR*) were then evaluated to identify specific isolates contributing significantly to the results. For the analysis of host sex (male vs. female) in relation to the occurrence of MDR/XDR profiles, Fisher’s Exact Test (2 × 2 table) was employed. Statistical analysis was performed using JASP (Version 0.96.0; JASP Team 2026, Amsterdam, The Netherlands). A *p*-value < 0.05 was considered statistically significant. Since multiple bacterial isolates could originate from the same animal (co-colonization), statistical analyses were performed at the isolate level. Consequently, the assumption of independence required for standard inferential tests was only partially satisfied due to potential clustering effects within individual hosts. This limitation was carefully considered when interpreting the significance of the associations, which should be interpreted as exploratory.

## 3. Results

### 3.1. Sampling Area

A total of 82 nasal swabs were collected from six hunting districts within the Avellino Province (Figure 2). Specifically, 16 nasal samples were obtained from the AVMFS009 Medio Fiume Sabato District (Venticano/Pietradefusi); 19 from the AVAR009 Arianese District (Bonito-Grottaminarda); 14 from the AVCP006 Picentini District (Chiusano); 18 from AVMFS005 Medio Fiume Sabato District (Prata di Principato Ultra); 8 from AVCP003 Picentini District (Montella); and 7 from AVSA009 Sant’Angelo District (Lioni). The sampled population consisted of 43 males (52%) and 39 females (48%). Regarding age distribution, juveniles (0–12 months) were the most represented class with 38 animals (46.3%), followed by subadults (13–36 months) with 24 animals (29.3%), and adults (>36 months) with 20 animals (24.4%).

### 3.2. Isolation and Identification of Species of the Genus Streptococcus

**Figure 3:**
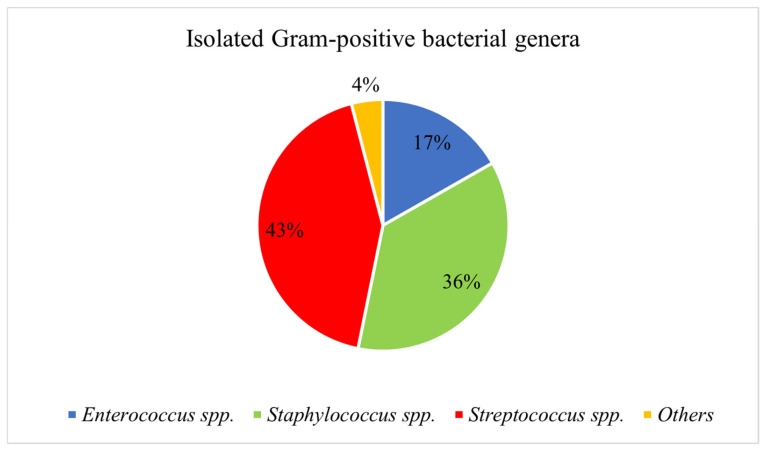
Identification frequency of Gram-positive bacterial genera.

A total of 173 Gram-positive bacterial isolates were recovered from 82 nasal swabs. Among these, 74 isolates were identified as *Streptococcus* spp., originating from 59 positive swabs (72%; 59/82; 95% CI: 60.9–81.3%). Regarding the Gram-positive nasal microbiota, *Streptococcus* spp. was the most frequent genus identified, accounting for 43% (74/173; 95% CI: 35.3–50.5%) of the isolates, as illustrated in Figure 3. The second most frequent genus was *Staphylococcus* spp., representing 36% (63/173; 95% CI: 29.3–44.0%) of the isolates, followed by *Enterococcus* spp. at 17% (30/173; 95% CI: 12.0–24.0%). The remaining 4% (6/173; 95% CI: 1.3–7.4%) consisted of other Gram-positive bacterial genera detected at lower frequencies.

**Figure 4:**
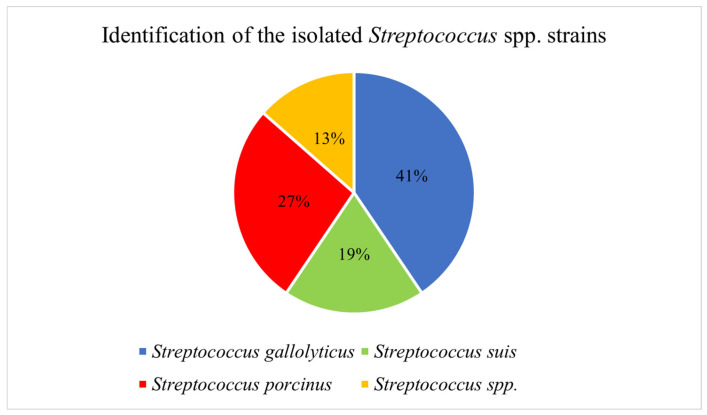
Identification frequency of *Streptococcus* species isolated from nasal cavities of wild boars.

As shown in Figure 4, among *Streptococcus* spp. isolated from wild boar nasal cavities, *Streptococcus gallolyticus* was the most frequently detected species (41%; 30/74; 95% CI: 29.3–52.5%), followed by *Streptococcus porcinus* (27%; 20/74; 95% CI: 17.6–38.6%) and *Streptococcus suis* (19%; 14/74; 95% CI: 10.8–29.7%). Additional species identified in smaller numbers included *Streptococcus pluranimalium* (7%; 5/74; 95% CI: 2.2–15.1%), *Streptococcus infantarius* (3%; 2/74; 95% CI: 0.3–9.4%), *Streptococcus dysgalactiae* (3%; 2/74; 95% CI: 0.3–9.4%), and *Streptococcus lutetiensis* (1%; 1/74; 95% CI: 0.0–7.3%). MALDI-TOF MS log(score) values for all *Streptococcus* species isolates ranged between 2.2 and 2.3, confirming highly probable species-level identification.

Co-colonization by different *Streptococcus* species within the same animal was observed in 15 out of 59 positive swabs (25%; 95% CI: 15.3–38.3%). The most frequent combination was the association of *Streptococcus porcinus* and *Streptococcus gallolyticus* (47%; 7/15; 95% CI: 21.3% –73.4%), followed by the association of *Streptococcus suis* and *Streptococcus gallolyticus* (27%; 4/15; 95% CI: 7.8–55.1%).

Regarding spatial distribution, no statistically significant association was found between sampling districts and species occurrence (χ^2^ = 14.46, *df* = 15, *p* = 0.491), with a Cramer’s V of 0.255. Descriptively, *Streptococcus gallolyticus* was most frequently detected in Bonito-Grottaminarda (55%; 11/20; 95% CI: 31.5–76.9%) and Venticano/Pietradefusi (50%; 6/12; 95% CI: 21.1–78.9%), whereas *Streptococcus suis* showed its highest occurrence in Lioni (67%; 2/3; 95% CI: 9.4–99.2%). *Streptococcus porcinus* predominated in Chiusano (37%; 7/19; 95% CI: 16.3–61.6%), while other identified *Streptococcus* species were detected at lower frequencies across districts. The isolation frequency of *Streptococcus* species across the six sampling districts is detailed in Table 2.

### 3.3. Antimicrobial Resistance Profiles of Identified Streptococcus spp.

**Figure 5:**
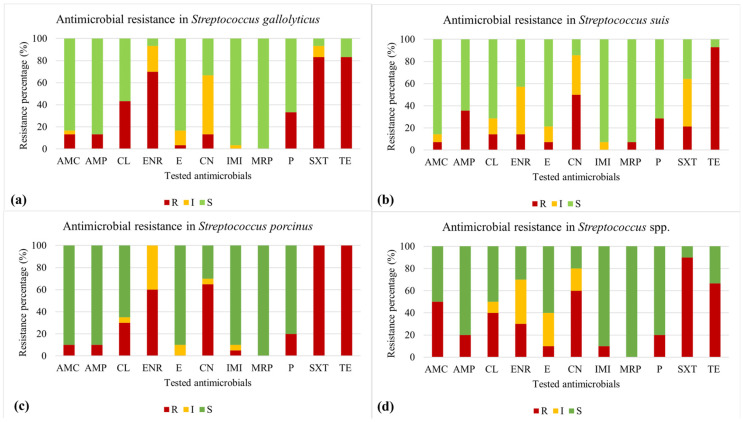
Antimicrobial resistance profiles of the identified *Streptococcus* spp. isolates. (**a**) *Streptococcus gallolyticus* (*n* = 30); (**b**) *Streptococcus suis* (*n* = 14); (**c**) *Streptococcus porcinus* (*n =* 20); (**d**) other identified *Streptococcus* spp. (*n* = 10). Tested antimicrobials: AMC: amoxicillin/clavulanic acid; AMP: ampicillin; CL: cephalexin; ENR: enrofloxacin; E: erythromycin; CN: gentamicin; IMI: imipenem; MRP: meropenem; P: penicillin; SXT: sulfamethoxazole-trimethoprim; TE: tetracycline.

The antimicrobial resistance profiles of the recovered *Streptococcus* species showed marked variability across the tested antimicrobials (Figure 5). All *Streptococcus porcinus* isolates (100%; 20/20; 95% CI: 83.2–100%) were resistant to both sulfamethoxazole–trimethoprim (SXT) and tetracycline (TE). High resistance to TE was also observed in *Streptococcus suis* (93%; 13/14; 95% CI: 66.1–99.8%) and *Streptococcus gallolyticus* (83%; 25/30; 95% CI: 65.3–94.4%). Similarly, elevated SXT resistance frequencies were recorded for *Streptococcus gallolyticus* (83%; 25/30; 95% CI: 65.3–94.4%) and the other identified *Streptococcus* spp. group (90%; 9/10; 95% CI: 55.5–99.7%). Furthermore, high levels of intermediate susceptibility were noted in *Streptococcus suis* for enrofloxacin (ENR) and SXT (43%; 6/14; 95% CI: 17.7–71.1%), as well as gentamicin (CN) (36%; 5/14; 95% CI: 12.8–64.9%). Intermediate susceptibility was also identified in *Streptococcus gallolyticus* for CN (53%; 16/30; 95% CI: 34.3–71.7%) and in *Streptococcus porcinus* for ENR (40%; 8/20; 95% CI: 19.1–63.9%).

### 3.4. Occurrence of Multidrug- and Extensively Drug-Resistant Streptococcus Isolates

Based on their antimicrobial susceptibility profiles, the recovered isolates were further classified according to multidrug resistance phenotype. Overall, 69% (51/74; 95% CI: 57.1–79.2%) of the isolates were classified as MDR, whereas 5% (4/74; 95% CI: 1.5–13.3%) were identified as XDR. No PDR isolates were detected. The highest MDR isolation frequency was observed in *Streptococcus porcinus* (85%; 17/20; 95% CI: 62.1–96.8%), followed by *Streptococcus gallolyticus* (73%; 22/30; 95% CI: 54.1–87.7%), and the other identified *Streptococcus* spp. group (70%; 7/10; 95% CI: 34.8–93.3%). Conversely, MDR phenotypes were less frequent in *Streptococcus suis*, accounting for 36% (5/14; 95% CI: 12.8–64.9%) of the isolates. XDR strains were sporadically identified, with one isolate each detected in *Streptococcus gallolyticus* (3%; 1/30; 95% CI: 0.1–17.2%) and *Streptococcus porcinus* (5%; 1/20; 95% CI: 0.1–24.9%), and two in the other identified *Streptococcus* spp. group (20%; 2/10; 95% CI: 2.5–55.6%). No XDR isolates were recovered from *Streptococcus suis*.

Chi-squared analysis revealed a significant association between bacterial species and the combined MDR/XDR resistance profile (χ^2^ = 16.70, *df* = 3, *p* < 0.001), with a Cramer’s V effect size of 0.472. Analysis of standardized residuals (*SR*) identified *Streptococcus suis* as the species with the lowest occurrence of MDR/XDR phenotypes within the studied isolates (*SR* = −3.92). In contrast, *Streptococcus porcinus* contributed positively to the distribution of multidrug resistance (*SR* = 1.97), while *Streptococcus gallolyticus* and the other identified *Streptococcus* spp. group showed lower positive deviations toward multidrug resistance (*SR* = 0.53 and *SR* = 1.28, respectively).

Furthermore, host-related factors did not appear to influence the distribution of resistance. No significant association was observed between host sex and the occurrence of MDR/XDR strains (*p* = 0.325), with MDR/XDR phenotypes detected in 76% (26/34) of males and 88% (22/25) of females. Similarly, age categories did not significantly affect the distribution of resistance profiles (*χ*^2^ = 2.315, *df* = 2, *p* = 0.314), with MDR/XDR observed at 83% (25/30) in the 0–12 months group, 67% (8/12) in the 13–36 months group, and 88% (15/17) in the >36 months group.

## 4. Discussion

To the best of our knowledge, this is the first study in Italy specifically aimed at characterizing *Streptococcus* species and their antimicrobial resistance profiles from the nasal cavities of wild boars hunted in the Avellino Province, Southern Italy. *Streptococcus* spp. were prioritized in this investigation due to their high prevalence in the nasal microbiota of the sampled animals. Therefore, the present study addresses a current knowledge gap by providing specific data on the presence and relative abundance of *Streptococcus* spp. in wild boar nasal samples.

Specifically, in our study, the detection of *Streptococcus gallolyticus*, *Streptococcus porcinus*, and *Streptococcus suis* suggests that the upper respiratory tract of wild suids represents an important ecological niche for diverse streptococcal species, in addition to *Staphylococcus* spp., *Enterococcus* spp. and *Escherichia coli*, as recently reported [25,26]. Although these three species are all Gram-positive cocci belonging to the genus *Streptococcus*, they occupy distinct ecological and clinical niches.

*Streptococcus suis* is a major zoonotic swine pathogen; in domestic pigs, the nasal cavity and tonsils are recognized as primary colonization sites where it can be carried asymptomatically while maintaining the potential to cause systemic disease under favorable conditions [27]. Consequently, wild boars may represent a noteworthy component in the ecology and potential transmission dynamics of this agent. Its detection in free-ranging populations is of significant epidemiological importance, as these animals could facilitate environmental persistence and potentially act as natural reservoirs within transmission cycles [15,17]. Notably, the bacterium has been identified even in the absence of associated lesions, further highlighting the role of wild boars in the silent maintenance of the pathogen [15]. From a One Health perspective, the identification of *Streptococcus gallolyticus* in nasal samples is also noteworthy. Although typically a gastrointestinal commensal in mammals, including swine, this Lancefield group D *Streptococcus* (*Streptococcus bovis* complex) can act as an opportunistic pathogen under specific conditions [28]. Its detection outside the intestinal tract may indicate transient colonization, environmental contamination, or broader mucosal distribution in wildlife hosts than currently recognized. Finally*, Streptococcus porcinus*, a *β*-hemolytic *Streptococcus* species (not yet classically grouped), although less studied than *Streptococcus suis*, has been reported among atypical streptococci capable of causing systemic infections in pigs, including septicemia and meningitis [29]. Our findings of *Streptococcus porcinus* in nasal cavities of wild boars may suggest either commensal colonization or early-stage carriage, although epidemiological data in wildlife remain extremely limited.

Beyond the microbiological characterization, the spatial distribution of these species was also evaluated. In the present study, no statistically significant association was found between sampling districts and species occurrence (*χ*^2^ = 14.46, *p* = 0.491). Although this result suggests the absence of marked spatial variation within the analyzed sample, it should not be interpreted as conclusive evidence of a homogeneous epidemiological distribution across the province. Rather, these non-significant findings indicate only the absence of statistically detectable geographic clustering within the constraints of the present study design. Some limitations should be acknowledged. The relatively small sample sizes in specific locations, such as Montella (AVCP003 Picentini District) and Lioni (AVSA009 Sant’Angelo District), limited the statistical power to detect subtle geographic differences. Furthermore, this study utilized an opportunistic sampling strategy, as samples were collected exclusively from animals harvested during the official hunting season. This may have introduced selection bias, meaning the sampled animals may not fully represent the broader wild boar population. Second, because the statistical analysis was conducted at the isolate level rather than animal level, potential clustering effects from multiple isolates per animal must be considered when interpreting inferential estimates and the significant associations should be interpreted as exploratory.

Despite these limitations, the simultaneous detection of multiple *Streptococcus* species, such as *Streptococcus gallolyticus*, *Streptococcus porcinus* and *Streptococcus suis*, highlights the complexity of the upper respiratory microbiota in wildlife. These findings may further highlight the potential function of wild boars as an interface species between wildlife ecosystems, domestic pigs, and humans. Given the zoonotic potential of several swine-associated streptococci, nasal carriage in wild boars could represent a potential source of occupational exposure for hunters, veterinarians, and individuals involved in carcass handling.

The antimicrobial resistance levels observed in this study may indicate a potential reduced susceptibility to several antimicrobial agents among the recovered isolates. However, as data on direct antimicrobial exposure history were not available, the specific drivers of these resistance phenotypes cannot be definitively determined. The high resistance levels to tetracycline and sulfamethoxazole-trimethoprim, notably the resistance in all *Streptococcus porcinus* (100%), the high frequency in *Streptococcus suis* (93%) and *Streptococcus gallolyticus* (83%), may reflect multiple, non-mutually exclusive factors. These include hypothesized environmental exposure to residues, indirect contact with livestock-associated bacterial populations, species-specific susceptibility profiles, or intrinsic reduced susceptibility to certain antimicrobial classes.

In *Streptococcus* species, tetracycline resistance is frequently linked to transferable determinants such as *tet*M, *tet*O, and *tet*S, which are widely disseminated across the genus [30]. While *tet*M gene has been reported in up to 93% of tetracycline-resistant isolates in literature [31], no molecular characterization was performed in the present study; therefore, the specific genetic basis of the observed phenotypes remains to be elucidated. From an epidemiological perspective, these levels appear consistent with historical reports of antimicrobial use in animal production [32], which could have contributed to the long-term maintenance of resistance genes in bacterial reservoirs even after regulatory restrictions [31]. Beyond individual resistance profiles, the frequency of multidrug resistance observed in this study (69%) deserves attention. This finding suggests that a substantial proportion of *Streptococcus* isolates circulating in the wild boar population exhibited resistance to at least three different antimicrobial classes. The detection of XDR profiles (5%), although at a relatively low frequency, further highlights the presence of isolates with limited therapeutic options. While no PDR strains were identified, these findings remain relevant from both clinical and epidemiological perspectives. Our results are consistent with previous studies reporting the widespread occurrence of MDR bacteria in wild boar populations and other wildlife species, supporting the hypothesis of free-ranging animals as potential environmental sentinels for antimicrobial resistance dissemination [3,33]. In particular, comparable MDR prevalence has been described in bacterial isolates from wild boars in Southern Italy, further suggesting a possible influence of anthropogenic pressures at the wildlife–livestock interface [25,26].

In the present study, Chi-squared analysis and standardized residuals confirmed that the occurrence of resistance profiles is significantly associated with the bacterial species (*p* < 0.001). Overall, these findings suggest that antimicrobial resistance in streptococci isolated from wild boars is associated with the specific bacterial species, with specific zoonotic pathogens displaying divergent resistance risks within the limits of this exploratory study. Specifically, *Streptococcus suis* was identified as the species with the lowest occurrence of MDR/XDR phenotypes (*SR* = −3.92), whereas *Streptococcus porcinus* contributed positively to the distribution of multidrug resistance (*SR* = 1.97). The occurrence of elevated levels of multidrug resistance in wildlife, which are generally not directly exposed to antimicrobial treatments, could hypothetically reflect environmental selective pressures or the horizontal transfer of resistance genes within the nasal microbiota [26]. These findings suggest a potential spill-over effect, whereby wildlife might mirror the environmental resistome shaped by anthropogenic activities and by ecological proximity to livestock production systems, highlighting the potential role of free-ranging animals as indicators of antimicrobial resistance circulation across the One Health interface [4,34], regardless of host demographics.

Resistance levels to enrofloxacin and gentamicin (approximately 60%), while lower than those for tetracycline, could also merit attention as these antimicrobials are often considered alternative therapeutic options when first-line drugs fail. These findings suggest a reduced phenotypic susceptibility, though the ecological factors contributing to these patterns, including potential co-selection phenomena, remain unclear. Resistance genes on mobile genetic elements often cluster, potentially facilitating the persistence of multidrug-resistant phenotypes even under selective pressure from a single antimicrobial class [33]. Importantly, the species-specific differences observed in resistance and intermediate susceptibility profiles might reflect the potential influence of species ecology, genetic plasticity, and hypothesized antimicrobial exposure history in shaping resistance evolution [33]. For instance, in *Streptococcus suis*, tetracycline resistance determinants such as *tet*O and *tet*W are widely documented, with a large proportion of isolates carrying at least one tetracycline resistance determinant, highlighting the extensive dissemination of these genes in animal-associated streptococci as reported in previous studies [35].

Another relevant finding is the high proportion of isolates showing intermediate susceptibility, such as in *Streptococcus suis* for enrofloxacin (43%) and in *Streptococcus gallolyticus* for gentamicin (53%). While these findings appear to align with global observations of antimicrobial resistance in various *Streptococcus* species [36], the descriptive nature of this study warrants a cautious interpretation of such trends, underlining the need for continued species-specific surveillance and antimicrobial stewardship.

Taken together, these results suggest that resistance phenotypes might be stabilized within the investigated bacterial populations, highlighting the potential importance of wild boars as potential sentinels for environmental antimicrobial resistance. Future investigations incorporating molecular characterization and longitudinal surveillance are necessary to better understand these dynamics and inform targeted stewardship interventions. Furthermore, the high resistance levels detected for multiple antimicrobial classes may compromise therapeutic options and facilitate the persistence and spread of resistant *Streptococcus* spp. strains within wildlife populations. This dissemination, potentially extending along the food chain, may pose significant veterinary and public health concerns.

## 5. Conclusions

In conclusion, this descriptive study highlights the presence of a variety of *Streptococcus* species in the nasal cavities of wild boars from Avellino Province in Southern Italy. The observed frequency of isolates with multidrug-resistant phenotypes, which showed a significant species-dependent distribution, suggests that these bacterial populations could potentially harbor diverse resistance determinants. However, given the exploratory nature of this study and the convenience sampling approach, these findings should be interpreted strictly as a preliminary snapshot of the local ecological context. While wild boars could potentially contribute to the environmental maintenance of antimicrobial resistance, further longitudinal studies incorporating molecular data are required to better understand the possible dynamics at the wildlife–livestock–human interface. Within a One Health framework, these results underline the importance of including wildlife in surveillance programs to better elucidate their potential contribution to the global burden of antimicrobial resistance.

## Figures and Tables

**Table 1:** Antimicrobial classes and agents tested for susceptibility profiling.

Antimicrobials	Disk Content	Antimicrobial Class	References for Breakpoints
Amoxicillin/Clavulanic Acid (AMC)	20/10 µg	Penicillins	[22]
Ampicillin (AMP)	10 µg		[22]
Penicillin (P)	10 IU		[22]
Cefalexin (CL)	30 µg	First-generation cephalosporins	[22]
Enrofloxacin (ENR)	5 µg	Fluoroquinolones	[22]
Gentamicin (CN)	10 µg	Aminoglycosides	[22]
Imipenem (IMI)	10 µg	Carbapenems	[23]
Meropenem (MRP)	10 µg		[23]
Erythromycin (E)	15 µg	Macrolides	[22]
Tetracycline (TE)	30 µg	Tetracyclines	[23]
Sulfamethoxazole-trimethoprim (SXT)	23.75/1.25 µg	Sulphonamides	[23]

**Table 2:** Distribution of *Streptococcus* species by sampling district.

Sampling District	*n*. Nasal Swabs	*Streptococcus gallolyticus* *n* (%)	*Streptococcus suis* *n* (%)	*Streptococcus porcinus* *n* (%)	Other *Streptococcus* spp. *n* (%)	Total Isolates (*N*) *
Bonito-Grottaminarda	19	11 (55%)	4 (20%)	4 (20%)	1 (5%)	20
Prata Principato Ultra	18	5 (42%)	1 (8%)	4 (33%)	2 (17%)	12
Venticano/Pietradefusi	16	6 (50%)	1 (8%)	3 (25%)	2 (17%)	12
Chiusano	14	5 (26%)	3 (16%)	7 (37%)	4 (21%)	19
Montella	8	3 (38%)	3 (38%)	1 (13%)	1 (13%)	8
Lioni	7	0 (0%)	2 (67%)	1 (33%)	0 (0%)	3
Total	82	30	14	20	10	74

* Percentages are calculated based on the total number of isolates recovered per location (*N*).

## Data Availability

The original contributions presented in this study are included in the article. Further inquiries can be directed to the corresponding authors.

## Abbreviations

The following abbreviations are used in this manuscript:

AMC	amoxicillin/clavulanic acid
AMP	ampicillin
BHI	Brain Heart Infusion broth
BTS	bacterial test standard
CL	cephalexin
CNA	Columbia CNA blood agar with 5% sheep blood
ENR	enrofloxacin
E	erythromycin
CN	gentamicin
IMI	imipenem
MDR	multidrug-resistant
MRP	meropenem
P	penicillin
PDR	pandrug-resistant
SXT	sulfamethoxazole-trimethoprim
TE	tetracycline
XDR	extensively drug-resistant
